# Predicting early response to ablative radiotherapy in oligometastatic disease: a scoping review of radiomics-based machine learning and deep learning models

**DOI:** 10.1007/s00330-026-12590-9

**Published:** 2026-04-30

**Authors:** Raquel García-Pablo, Marta Canela-Capdevila, Alberto Martínez-Caballero, Rocío Benavides-Villareal, Albert Moragas-Fernández, Andrea Jiménez-Franco, Berta Piqué-Smith, Camila Montesinos-Guevara, Jordi Camps, Jorge Joven, Angel Torrado-Carvajal, Meritxell Arenas

**Affiliations:** 1 Department of Radiation Oncology, Hospital Universitari de Sant Joan, Institut d’Investigació Sanitària Pere Virgili, Universitat Rovira i Virgili, Reus, Spain; 2 Unitat de Recerca Biomèdica, Hospital Universitari Sant Joan de Reus, Institut d’Investigació Sanitària Pere Virgili, Universitat Rovira i Virgili, Reus, Spain; 3 Medical Image Analysis and Biometry Laboratory, Universidad Rey Juan Carlos, Móstoles, Madrid, Spain; 4 Organisation for Economic Co-operation and Development (OECD) Health Division, Paris, France; 5 Cochrane Ecuador, Centro de Investigación en Salud Pública y Epidemiología Clínica (CISPEC), Facultad de Ciencias de la Salud Eugenio Espejo, Universidad UTE, Quito, Ecuador

**Keywords:** Stereotactic body radiotherapy, Deep learning, Machine learning, Metastases, Radiomics

## Abstract

**Objectives:**

Oligometastatic disease represents an intermediate stage of cancer, often treated with surgery or ablative radiotherapy (ART). This scoping review aimed to systematically summarize current evidence on the use of radiomics, including machine learning and deep learning approaches, to predict response to ART. We also aimed to assess the methodological quality and reporting transparency of published studies, identifying gaps and opportunities for future research.

**Materials and methods:**

A systematic search in PubMed, Web of Science, Scopus, Embase, Cochrane, and Google Scholar identified studies that used radiomics for predicting ART response. Two reviewers independently selected and assessed the methodological quality using the Radiomics Quality Score (RQS) and the METhodological RadiomICs Score (METRICS). In addition, reporting transparency was evaluated using the CheckList for EvaluAtion of Radiomics research (CLEAR). This scoping review follows the Preferred Reporting Items for Systematic Reviews and Meta-Analyses (PRISMA) extension for Scoping Reviews guidelines.

**Results:**

The systematic search identified 9463 records, of which 29 studies (3946 patients) were included. Most studies used MRI-derived features, with 24 focusing on brain metastases. Radiomics-based models demonstrated variable predictive performance (area under the curve, AUC: 0.69–0.95), with deep learning models achieving the highest accuracies (AUC: 0.85–1.00). Methodological quality of the studies was moderate (mean RQS: 13; METRICS: 64.2–78%).

**Conclusion:**

Radiomics-based models show potential for identifying patients unlikely to benefit from ART, but their clinical implementation remains limited, especially for extracranial metastases. Future research should focus on multicenter, prospective studies with standardized protocols, incorporating clinical and dosimetric data for broader clinical application.

**Key Points:**

***Question*** *Can radiomics-based predictive models reliably assess treatment response to ablative radiotherapy in oligometastatic disease, and how robust is the current methodological evidence supporting their use?*

***Findings*** *Radiomics models show encouraging predictive performance, mainly for brain metastases, yet substantial methodological heterogeneity and limited validation hinder their clinical translation.*

***Clinical relevance*** *Radiomics-based prediction models hold potential for identifying patients unlikely to benefit from ablative radiotherapy, enabling more personalized treatment. Further prospective, multicenter, and methodologically standardized studies are essential before clinical implementation.*

**Graphical Abstract:**

**Figure:**
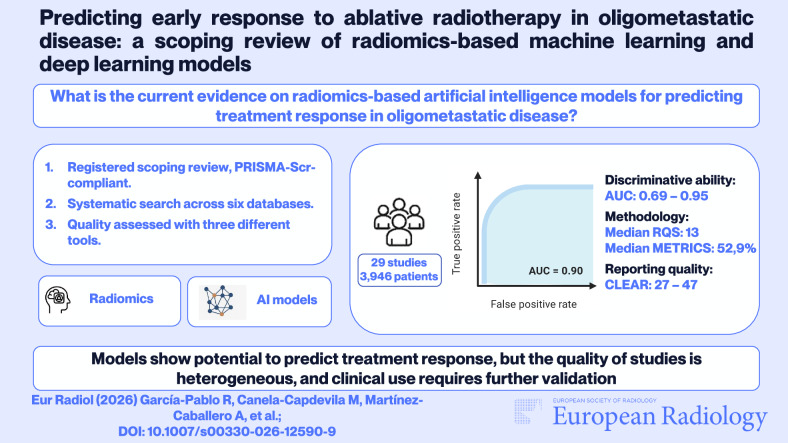

## Introduction

Cancer remains one of the leading causes of death worldwide, with an estimated 9.7 million deaths in 2022 [1]. The concept of oligometastatic disease (OMD), introduced by Hellman and Weichselbaum in 1995 [2], represents an intermediate state between localized and widespread cancer. According to the European and American Societies for Radiotherapy and Oncology, OMD involves one to five safely treatable metastases that are smaller than 5 cm. In such cases, radical local treatment may achieve long-term control and ultimately lead to a cure [3].

OMD management relies mainly on local treatments such as surgery and ablative radiotherapy (ART) [4, 5]. While surgery remains the preferred approach, many patients are unsuitable candidates; in such cases, ART offers a non-invasive, accurate alternative capable of eradicating small tumor foci while sparing surrounding tissue. Advances in imaging and planning have consolidated ART as a key component in OMD treatment. Stereotactic techniques include radiosurgery (SRS) and fractionated radiotherapy (SRT) for brain metastases. At the same time, extracranial lesions in sites such as bone, lung, liver, and lymph nodes are typically treated with stereotactic body radiotherapy (SBRT). The choice of technique depends on lesion size, location, proximity to critical structures, and institutional protocols [4–14].

Despite technological progress, ART failure rates reach up to 30% in the brain [6] and 30–40% in extracranial lesions [15, 16]. Therefore, identifying patients at higher risk of local failure is crucial to guide personalized strategies. Radiomics, which extracts quantitative features from imaging modalities such as MRI, CT, and PET, has emerged as a promising approach for capturing imaging biomarkers that reflect tumor biology [17]. Artificial intelligence (AI), particularly machine learning (ML) and deep learning (DL) techniques, can analyze these high-dimensional data to predict outcomes and support decision-making [17–19]. DL, based on neural networks, enables automatic feature extraction and often achieves superior predictive performance [18]. Integrating radiomics with clinical data may further enhance the robustness and accuracy of the model [20, 21].

Although ML and DL models based on radiomic data have shown promise in predicting early treatment response in metastatic lesions, most available studies are single-center and methodologically heterogeneous, limiting their generalizability and clinical applicability. To address this gap, we conducted a scoping review with two main objectives: (1) to systematically assess the predictive performance of ML and DL models using radiomic data alone or combined with clinical variables for evaluating early treatment response in metastatic lesions of OMD patients treated with ART; and (2) to evaluate methodological quality using the validated and complementary Radiomics Quality Score (RQS) [22], and the METhodological RadiomICs Score (METRICS) [23], and to analyze the reporting transparency using the CheckList for EvaluAtion of Radiomics research (CLEAR) [24, 25]. Our approach provides a robust framework to contextualize current evidence and its potential clinical applicability.

## Methods

The current review is registered in the Open Science Framework under the registration 10.17605/OSF.IO/FAWYE and was prepared in accordance with the Preferred Reporting Items for Systematic reviews and Meta-Analysis (PRISMA) Extension for Scoping Reviews [26]. Ethical approval and informed consent were not required, as only previously published data were analyzed and no individual patient information was used.

### Search strategy

A systematic literature search was performed using the Patient, Intervention, Comparator, and Outcome (PICO) strategy across six electronic databases: PubMed, Web of Science, Scopus, Embase Ovid, Cochrane Library, and Google Scholar. Keywords, controlled terminology (MeSH), and Boolean operators were used to maximize search sensitivity. The search strategy was prepared for each database using keywords such as “oligometastatic disease,” “brain metastases,” “bone metastases,” “lung metastases,” “liver metastases,” “stereotactic radiosurgery,” “SRS,” “SRT,” “stereotactic ablative radiotherapy,” “SABR,” “SBRT,” “radiomics,” “machine learning” and “deep learning.” The search started on 01/04/2024. The software Polyglot from Systematic Review Accelerator© [27] was used to translate the search strategy across databases. Search strategies were saved in various databases, and monthly alerts were set up for new publications (with an end date of June 12, 2025). The entire search strategy is provided in Supplementary Table 1.

### Eligibility criteria

There were no restrictions on publication date. Only articles written in English, French, or Spanish were included.

### Inclusion criteria

1. Patients with brain, bone, lung, or liver metastases treated with SRS/SRT, surgery + SRS/SRT, or SABR alone, and other oncological treatments.
2. ML or DL models based on radiomic features from CT, MRI, or PET to predict early radiologic treatment response within 2–6 months after ART (only one study evaluated at 12 months after ART).
3. Reported performance metrics of the models, including accuracy, specificity, sensitivity, and AUC of the ROC curves.
4. Cohort, cross-sectional, or case-control studies.

### Exclusion criteria

1. Non-human studies, studies involving pregnant women, or studies on patients younger than 18 years.
2. Studies with no radiomic data, or ML and DL models that did not aim to predict early treatment response.
3. Clinical practice guidelines, abstracts, letters to the editor, seminars, case series, systematic and narrative reviews, and meta-analyses.

### Study selection

After removing duplicates, two reviewers independently screened the studies retrieved from the literature search based on the predefined inclusion and exclusion criteria. Articles were first assessed by title and abstract, followed by full-text assessment. Discrepancies were resolved through consensus. The review process was performed using Rayyan©, an online software tool [28].

### Data extraction

Data extraction was performed using a Microsoft Excel© spreadsheet and included:

1. Study and patient characteristics: author, publication year, country, study design, study aim, number of patients and age, general patient status, primary tumor type, location and number of metastases, metastatic size, type of radiotherapy (SRS, SRT, or SABR), total dose, radiological response assessment, and follow-up.
2. Model evaluation: imaging modality, artificial intelligence software, number of features, region of interest, algorithms used, features included, and performance metrics.

### Assessment of radiomics methodological quality

The methodological quality of each radiomics study was evaluated using two validated frameworks: the RQS and METRICS. RQS comprises 16 items, with a total score ranging from −8 to +36, which is converted to a percentage using a non-linear scale (negative scores are set to 0%). METRICS is a comprehensive quality assessment framework with 30 items organized into nine categories, including five conditional elements to cover both handcrafted and DL radiomics approaches. It incorporates bias assessment, and scores are reported as percentages (0–100%) using a linear scaling method based on selected conditions. Assessments were conducted via METRICS’ dedicated online platform, which facilitates scoring according to consensus-derived item weights. Additionally, the CLEAR checklist comprises 58 items and outlines the minimum requirements for transparent reporting of clinical radiomics research. It is intended as a standardization tool rather than a quality score.

## Results

**Fig. 1:**
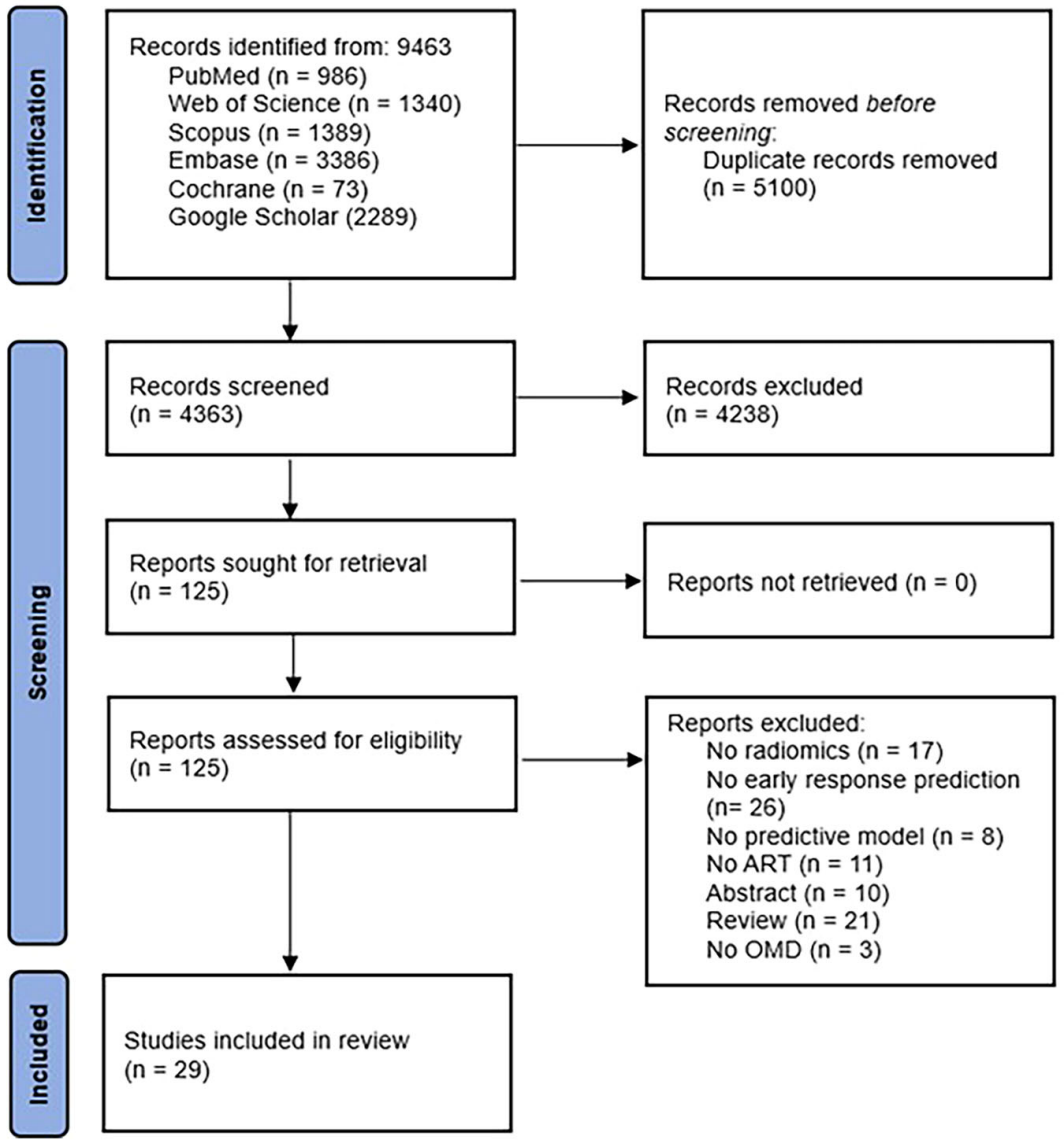
Flow diagram of the Preferred Reporting Items for Systematic Review and Meta-analysis (PRISMA). ART, ablative radiotherapy; OMD, oligometastatic disease

The literature search identified 9463 records across all the electronic databases. After removing 5100 duplicates and excluding 4238 studies based on title and abstract screening, 125 full-text articles were assessed for eligibility. Of these, 29 met the inclusion criteria defined by the PICO framework [15, 16, 18, 20, 29–53]. The complete study selection process is illustrated in Fig. 1, and detailed data from the 29 included studies are shown in Supplementary Tables 2 and 3.

### General characteristics of the studies

**Fig. 2:**
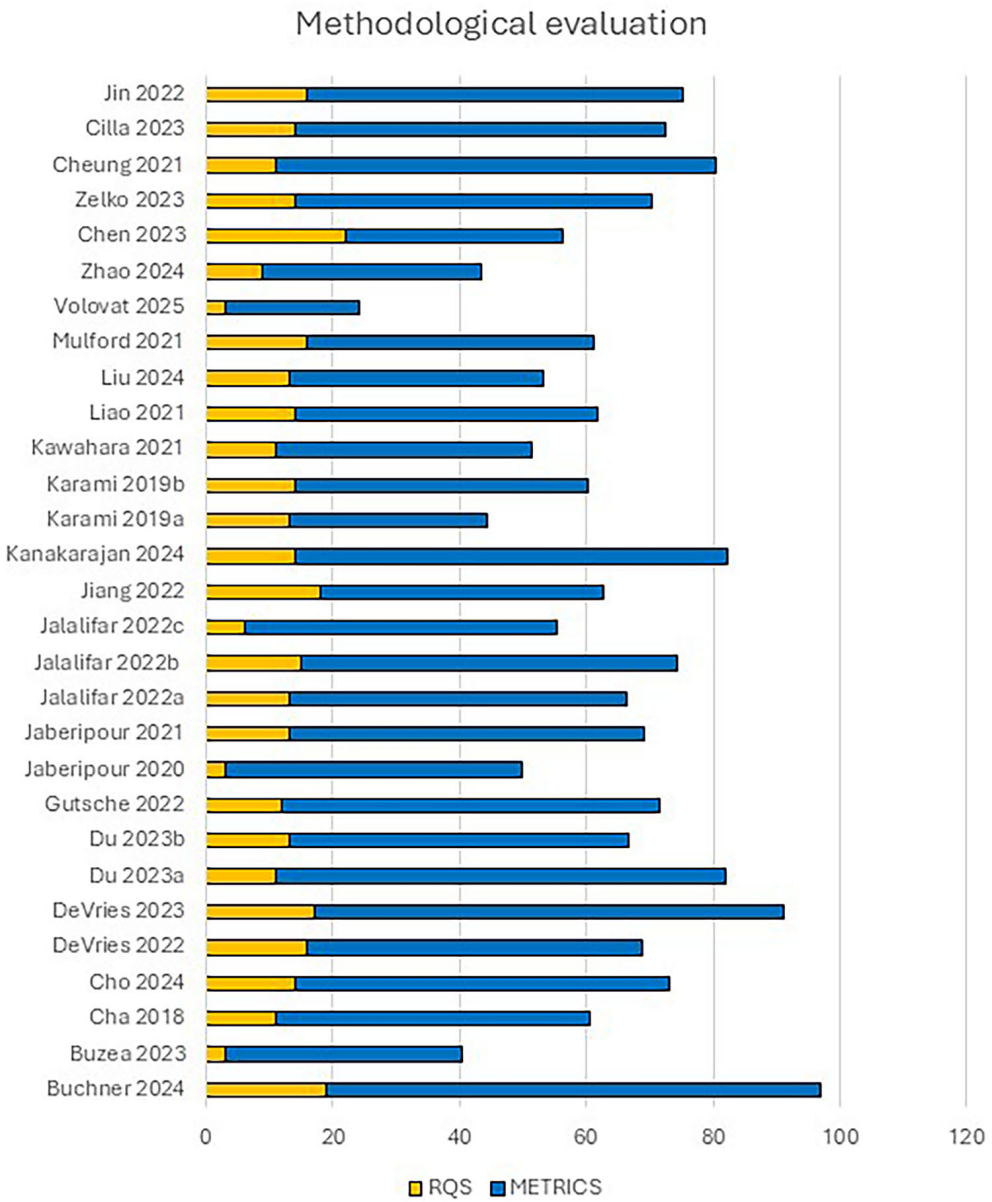
Methodological quality assessment of included studies. METRICS, METhodological RadiomICs Score; RQS, Radiomics Quality Score

A summary of the demographic and clinical characteristics of the patients included in the 29 selected studies is provided in Table 1. All studies were retrospective and published between 2018 and 2025, without sample size calculation, encompassing 3946 patients (interquartile range (IQR): 68–172) with Karnofsky Performance Status (KPS) score of 70 or higher and 6081 metastases (IQR: 112–274). Nevertheless, because some studies were conducted by the same authors, the possibility of overlapping patient cohorts and metastatic lesions across publications cannot be excluded (Fig. 2).

**Table 1:** General characteristics of the selected studies and their patients

Study	Patients, *n* (M/F)	General status	Metastases, *n*	Metastatic size (mL)	SABR method	Total dose (Gy/fx)	Scale	Early response evaluation
**Brain metastases**								
Buchner et al [29] 2024	352	KPS 80 (70–90)	NA	13 (5–24)	LINAC	20/1; 16/1 35/7; 30/6	NA	3 months
Buzea et al [30] 2023	19 (14/5)	KPS > 70	19	16 (2–81)	Gk	30/3	NA	NA
Cha et al [31] 2018	89	KPS = 90	110	2 (1–3.3)	CK	24/3; 23/3	RECIST	3 months
Cho et al [32] 2024	194 (103/91)	NA	369	NA	NA	NA	Modified RANO-BM	3 months
DeVries et al [33] 2022	99 (44/55)	ECOG 1 (0–1)	123	3.07 (0.02–30.23)	LINAC	15/1; 18/1; 21/1; 24/3	NA	3 months
DeVries et al [20] 2023	99 (44/55)	NA	240	3500 (100–41,200)	LINAC	21 (15–24)/1 21 (18–27)/1	RANO-BM	3 months
Du et al [34] 2023	307 (188/149)	KPS > 70	403	2.58 (0.39–51)	Gk	17 (15–20)/1	RANO-BM	2 months
Du et al [35] 2023	337 (186/151)	KPS > 70	501	3.24 (0.03–51)	LINAC	17 (15–20)/1	RANO-BM	2 months
Gutsche et al [36] 2022	150 (53/97)	NA	308	0.658 (0.024–13.667)	CK	20/1; 18/1; 27/3	RANO-BM	6 months
Jaberipour et al [37] 2020	120 (48/72)	NA	169	NA	LINAC	NA	RANO-BM	2–3 months
Jaberipour et al [38] 2021	120 (48/72)	NA	171	2 (0.4–7)	LINAC	22.5–35/5	RANO-BM	2–3 months
Jalalifar et al [39] 2022	124 (50/74)	NA	156	2 ± 1.03	NA	22.5–35	RANO-BM	2–3 months
Jalalifar et al [40] 2022	124 (50/74)	NA	156	2 ± 1.03	NA	NA	RANO-BM	2–3 months
Jalalifar et al [41] 2022	124 (50/74)	NA	156	2 ± 1.03	NA	NA	RANO-BM	2–3 months
Jiang et al [42] 2022	137	NA	213	2.24 (1.02–5.21)	Gk	60	RANO-BM	3 months
Kanakarajan et al [18] 2024	129 (54/75)	KPS 90 [80–100]	NA	17.44 (88–88.03)	Gk	22 (14–25)	NA	3 months
Karami et al [43] 2019	38	NA	38	NA	LINAC	NA	NA	3 months
Karami et al [44] 2019	100 (37/63)	NA	133	2.1 (0.4–7)	LINAC	30/5	RANO-BM	2–3 months
Kawahara et al [45] 2021	52 (28/24)	NA	157	< 4.2 4.2–14.1 > 14.1	Gk	24 Gy 18 Gy 15 Gy	Modified RECIST	3 months
Liao et al [46] 2021	256	NA	976	0–5 mm 5–10 mm 10–20 mm > 20 mm	Gk	28.6	NA	NA
Liu et al [47] 2024	337 (184/153)	80 (70–90)	960	6.82 (0.03–51)	NA	17 (15–20)	RANO-BM	NA
Mulford et al [48] 2021	67 (30/37)	NA	71 cavities	6.2 (0.2–31.6)	Gk	18 (15–22)	NA	2–3 months
Volovat et al [49] 2025	19 (14/5)	KPS = 70	NA	16 (2–81)	Gk	30/10	RANO-BM	NA
Zhao et al [52] 2024	82 (32/50)	NA	114	1.8 (0.4–5.4)	LINAC	NA	NA	3 months
**Bone metastases**								
Chen et al [16] 2023	194 (108/86)	KPS > 70	194	NA	CK	18–24/1; 24–30/3; 30–40/5	RECIST	NA
Zelko et al [51] 2023	130 (86/44)	NA	179	7.1 (0.06–178.9)	NA	12/2; 24/2; 25/5; 30/5; 35/5; 40/5	RECIST	6 months
**Lung metastases**								
Cheung et al [52] 2021	69 (42/27)	NA	85	NA	LINAC	54 (45–60)/3–5	RECIST	3 months
Cilla et al [15] 2023	56 (38/18)	NA	80	5.3 (0.2–61.9)	LINAC	50 (30–50)/5	RECIST	4 months
**Liver metastases**								
Jin et al [53] 2022	22	NA	NA	3.2 (1.3–8)	LINAC	30–60/1–5	RECIST and modified RECIST	12 months

Metastatic size is shown as median and interquartile range

*CK* Cyberknife, *ECOG* Eastern Cooperative Oncology Group, *F* female, *fx* fraction, *Gk* Gamma-knife, *Gy* Gray, *KPS* Karnofsky Performance Status, *LINAC* linear accelerator, *M* male, *mL* mililitres, *mm* milimetres, *NR* non-responders, *NA* not available, *R* responders, *RANO-BM* response assessment in neuro-oncology brain metastases, *RECIST* Response Evaluation Criteria In Solid Tumors

Most studies focused on brain metastases (*n* = 24; 82.7%), with a mean volume of 5.8 mL (range: 0.1–27.9 mL), while only a few investigated bone (*n* = 2; 6.3%), lung (*n* = 2; 6.3%), or liver lesions (*n* = 1; 3.1%). Metastases analyzed primarily arose from multiple primary cancers. The most reported primary tumor was lung cancer (*n* = 2146), followed by melanoma (*n* = 453), breast cancer (*n* = 428), colorectal cancer (*n* = 179), and renal cancer (*n* = 136). Additionally, two studies (6.3%) incorporated not only metastatic lesions but also primary tumor images from the same organ into their predictive models.

Regarding treatment modalities, LINear ACcelerator (LINAC)-based stereotactic radiotherapy was the most frequently used (*n* = 12; 37.5%). This technique uses LINAC to deliver highly focused radiation beams. Gamma Knife SRS, which employs multiple cobalt sources (up to 60) to target intracranial lesions with submillimeter precision, was reported in eight studies (27.5%). CyberKnife was described in 3 studies (9.4%) and consists of a robotic system capable of delivering SRT to both cranial and extracranial sites [54]. Six studies (19.4%) did not specify the treatment modality. Radiological response was assessed between 2 and 6 months after treatment. Only one was evaluated at 12 months after treatment (see Table 2). Administered doses ranged from 12 to 60 Gy, and response evaluation was performed using the Response Assessment in Neuro-Oncology Brain Metastases (RANO-BM) [54] or Response Evaluation Criteria In Solid Tumors (RECIST) [55] criteria, with or without modifications, in 16 and 7 studies, respectively [56].

**Table 2:** Methodological details of the selected studies

Study	Imaging modality	AI software	ROI	Algorithm	Features	Accuracy (%)	AUC/ROC (95% CI)	F1-Score (%)
**Brain metastases**								
Buchner et al [29] 2024	MRI	PyRadiomics	T + E	ENR	HC, Clinical		77 (61–87)	
Buzea et al [30] 2023	MRI	Python	NA	CNN	DL	P: 98 R: 98	98.9	P: 97 R: 99
Cha et al [31] 2018	CT	TensorFlow	T	CNN	HC		85.9 (68.2–100)	
Cho et al [32] 2024	MRI	Python	T + M	CNN	HC, DL, Dose	87.8	87.8	
DeVries et al [33] 2022	MRI	PyRadiomics	T	RF	HC, Clinical		69	
DeVries et al [20] 2023	MRI	PyRadiomics	T	RF	HC, Clinical		71 ± 1	
Du et al [34] 2023	MRI	PyRadiomics	T + E	SVM	HC, Clinical	80	93	77
Du et al [35] 2023	MRI	PyRadiomics	T + E	SVM	HC, Clinical	89	90	83
Gutsche et al [36] 2022	MRI	PyRadiomics	T + E	RF	HC, MRI semantic	75	74	
Jaberipour et al [37] 2020	NC-CT	PyRadiomics	T + E	AdaBoost	HC	71	72	
Jaberipour et al [38] 2021	MRI	PyRadiomics	T + E	k-NN	HC	87	87	
Jalalifar et al [39] 2022	MRI	Python	T + E	CNN + LSTM	DL, Clinical	82.5	86	
Jalalifar et al [40] 2022	MRI	PyRadiomics	T + M	SVM + Gk	HC	A: 80 MA: 80	A: 78 MA: 81	A: 76.5 M: 77.8
Jalalifar et al [41] 2022	MRI	Python	T + E + M	3DResNet + SelfAtt + BiT	DL	82.5	91	80
Jiang et al [42] 2022	MRI	PyRadiomics	T + E	RF	HC		84.8	
Kanakarajan et al [18] 2024	MRI	PyRadiomics	T	RF	HC, DL, Clinical	81.7 (77.7–85.6)	88 (85–91)	86.44 (83.3–89.6)
Karami et al [43] 2019	MRI	3D Slicer	T + E	SVM + Gk	HC	82	80	
Karami et al [44] 2019	MRI	3D Slicer	T + E + M	SVM	HC	80	82	
Kawahara et al [45] 2021	MRI	IBEX	T	NN	HC	78	87	
Liao et al [46] 2021	MRI	MR radiomics platform	T	SVM	HC, Clinical	89 ± 11	95 ± 9	
Liu et al [47] 2024	MRI	NVIDIA	T + E	SRTRP-Net	DL, Clinical	85.6	92.5	
Mulford et al [48] 2021	MRI	PyRadiomics	C	Gboost	HC	76	73 ± 8	72
Volovat et al [49] 2025	MRI	NA	T + E	ViT	DL	99	1	99
Zhao et al [50] 2024	MRI	NA	T + M	VGG-19	DL, Dose, Clinical	84 ± 8	89 ± 2	
**Bone metastases**								
Chen et al [16] 2023	MRI	uAI Research Portal platform 1.1	T	GP	HC, Clinical	83	82.8	
Zelko et al [51] 2023	CT	PyRadiomics	T + M	SVM	HC, Dose, Clinical		77 (64–89)	
**Lung metastases**								
Cheung et al [52] 2021	NC-CT	PyRadiomics	T	SVM	HC	74.8	85.7 (78.9–91.5)	72
Cilla et al [15] 2023	CT	PyRadiomics	T	CART	HC, Clinical	75 (71.7–78.3)	75.3 (67.5–83.6)	69.4 (66.8–71.9)
**Liver metastases**								
Jin et al [53] 2022	MRI	MATLAB	T	RF	HC, Clinical		89.5 (76.2–100)	

*3DResNet+SelfAtt+BiT* 3D residual network + self attention + big tansfer, *A* automated, *AdaBoost* adaptive boosting, *AlexNet + TLN* AlexNet deep learning + Transfer learning, *AI* artificial intelligence, *AUC* area under the curve, *C* cavity, *CART* classification and regression tree analysis, *CT* computed tomography, *DL* deep learning, *E* edema, *CNN* convolutional neural network, *ENR* Elastic Net Regularization, *Gboost* gradient boosting, *Gk* Gaussian kernel, *GP* Gaussian processes, *HC* handcrafted, *k-NN* k-nearest neighbor, *LR* logistic regression, *LSTM* long short-term memory, *M* margin, *MA* manually, *MRI* magnetic resonance imaging, *NA* not available, *NC-CT* non-constrast computed tomography, *NN* neuronal network, *P* progression, *R* regression, *RF* random forest, *ROC* receiver operating characteristics, *ROI* region of interest, *SRTRP-Net* SRS response prediction subnet, *SVM* support vector machine, *T* tumor, *VGG-19* visual geometry group, *ViT* vision transformers

### Predicting treatment response

The main methodological characteristics of the 29 selected studies are shown in Table 2. The most commonly used imaging modality was MRI, employed by 24 studies (82.7%), primarily for brain metastases (*n* = 22). CT images were also used in five studies (15.6%), although brain metastases were assessed exclusively with non-contrast CT, and the type of CT was not specified for non-spinal bone and lung metastases. Regarding data-splitting methodology, most authors used a randomized technique [36–41, 46, 53] and only a few used a manual approach [42, 45] or did not specify their method [29, 30, 32].

Given that most studies faced class imbalance issues, oversampling methods were commonly applied, with the synthetic minority oversampling technique (SMOTE) being the most frequently used [18, 29, 30, 40]. Most studies (*n* = 15; 51.7%) used PyRadiomics for radiomic feature extraction, followed by custom Python scripts (*n* = 4; 13%) and other software (*n* = 8; 25.0%). Two studies (6.3%) did not report the software employed. Across studies, a median of 256 (IQR: 107–1651) features per region of interest were extracted. Feature extraction was most commonly performed from the tumor region and its surrounding edema (*n* = 11, 37.9%), although 10 studies (34.4%) focused solely on the tumor region, and six (18.8%) also incorporated tumor edema into their analysis. One study (3.1%) extracted features from the resection cavity, and another (3.1%) did not specify the region.

The use of handcrafted radiomic features predominated (*n* = 22; 75.8%) over DL-derived features (*n* = 5; 15.6%), and only two authors combined both approaches. None of the extracranial metastases used DL. Clinical variables were incorporated in eight studies (27.5%), but radiation dose information was considered in only two (6.8%). To reduce the risk of model overfitting, feature reduction techniques were applied in most studies (*n* = 24; 82.7%), being Least Absolute Shrinkage and Selection Operator (LASSO) [45], minimum redundancy—maximum relevance (mRMR) [29, 38, 40], or both [34, 35, 53] the most commonly used approaches. Two studies (6.3%) did not perform any reduction, and three (10.3%) did not provide sufficient details about their methodology. A variety of ML algorithms were applied, with Support Vector Machine (SVM) being the most frequently used (*n* = 8; 25.0%), followed by Random Forest (RF) (*n* = 6; 20.6%) and Convolutional Neural Networks (CNN) (*n* = 5; 15.6%). For model validation, the most frequent methods were k-fold cross-validation [15, 16, 18, 36, 37, 40, 42, 45, 57], followed by bootstrap [18, 36].

When predicting the response of brain metastases to ART, radiomic models exhibited highly variable predictive performance, with reported AUC values ranging from 0.69 to 0.95. DL models showed higher accuracy, achieving AUC values between 0.85 and 1.00 and better F1-scores.

The inclusion of clinical variables consistently enhanced the predictive performance of the models. Key features incorporated into these models included KPS, tumor histology, metastatic site, lesion size or volume, number of metastases, prior treatments, and patient age.

For non-brain metastases, predictive performance data were limited due to the small number of studies. Nonetheless, similar levels of accuracy were reported, with median AUC values of 0.80 for bone metastases and 0.85 for lung metastases.

### Methodological and reporting quality

The mean RQS across the studies was 13, with the highest score being 22 [29]. Most studies reported a well-documented imaging protocol (*n* = 16; 55.1%) and performed internal validations of their predictive models (*n* = 24; 82.7%).

However, more than 90% of the studies scored negatively in the domains of “imaging at multiple time points,” “cutoff analyses,” and “open science and data sharing” (only eight studies (27.5%) shared their code). No points could be awarded in categories such as “phantom study across all scanners,” “identification and discussion of biological correlates,” “prospective study registered in a trial registry,” or “cost-effectiveness analysis.”

Moreover, none of the studies compared their predictive models against a universally accepted gold standard, mainly because such a benchmark is not defined within the RQS framework. In principle, biopsy of the metastasis would provide the most reliable reference for evaluating treatment response, but this is rarely performed in clinical practice.

Therefore, modest RQS scores reflect not only methodological limitations but also the fact that several RQS items capture practices that are still uncommon in radiomics research. The detailed RQS results are depicted in Supplementary Table 4.

Most studies were classified as having “moderate” quality, with METRICS values ranging from 40.2 to 59.5%. Five studies were rated as “low” quality (21–37%), while six achieved “good” quality scores (64.2–78%). Notably, all good-quality studies were published between 2023 and 2024. The complete METRICS evaluation is shown in Supplementary Table 5.

Analysis of the METRICS domains showed that methodological rigor is relatively robust in most radiomics studies, particularly in the reporting of performance evaluation metrics, the use of internal validation, and the description of preprocessing techniques. In contrast, aspects crucial for clinical implementation and generalizability remain underdeveloped as reflected by poor adherence to radiomic guidelines, limited use of multicenter designs, reduced clinical translatability, and restricted data or model availability, among others.

CLEAR scores ranged from 27 [38] to 47 [18, 29, 35] out of 58 points, with the highest scores corresponding to studies published since 2023, which have shown improved adherence to transparency in reporting. The complete CLEAR checklist is presented in Supplementary Table 6.

## Discussion

This scoping review evaluated studies using radiomics combined with ML and DL to predict early radiological response of brain, bone, lung, and liver metastases treated with ART. Methodological quality was assessed using RQS and METRICS, while transparency and reporting of predictive models were evaluated using CLEAR. Radiomics is a promising prognostic tool; however, the main methodological shortcomings continue to hinder its clinical translation. Most studies were retrospective, single-center, and highly heterogeneous, limiting cross-study comparability.

Most studies focus on brain metastases, with only six addressing extracranial lesions [15, 16, 53, 54, 57]. These imbalanced findings may reflect the fact that rescuing non-responding lesions in those areas is relatively easier to manage. Nevertheless, not all patients treated with SABR or SRS can undergo surgery due to difficult lesion access or patient frailty [4, 5]. Additionally, further studies with a large sample size are highly necessary to address the ART response of those patients unable to undergo surgery.

Patient cohorts were often small to moderate, often without a priori sample size calculations, limiting model generalizability, increasing overfitting risk and challenging the robustness of model development.

The radiomics workflow varied substantially across studies. Brain metastases studies mostly focused on MRI-derived features, but models using simulation CT–based features performed similarly, suggesting they are also suitable for predictive modeling. Additionally, extracranial studies focused on both image source types. Further studies are needed to contrast the predictive performance of MRI and CT-derived features in each type of lesion.

Most studies define “early response” at 2–3 months; however, one study assesses it at 4 months, two at 6 months, and one at 12 months (see Table 1). This variability in assessment time points results in non-uniform endpoints and may partially account for the differences in model performance observed across studies. Also, there is still no consensus on the optimal ROI definition. Considering edema and peritumoral tissue may reflect crucial microenvironmental processes for metastatic development and growth [37, 41–43, 50], but they could also introduce potential noise or redundant features to the model [37, 38]. High-quality brain metastases studies included edema [29, 34–36, 47] and reported a higher median AUC of 87.7 (IQR: 77–92.5), whereas extracranial studies generally focused only on the tumor lesion itself. Methodological differences highlight the need for further investigation.

The best approach for ROI segmentation is still uncertain. Most studies, including high-quality ones, relied on manual segmentation, which is generally more accurate but time-consuming. Only two studies applied automatic segmentation [42, 47], which offers advantages in efficiency and reproducibility [19] but may introduce errors affecting model outcomes. Moreover, the effect of minor segmentation differences remains debated: while Jalalifar et al reported minimal influence on model outcomes [40], Jin et al found substantial impact on radiomic feature extraction [54]. Future research should focus on standardizing segmentation methods and systematically assessing how methodological variations affect predictive accuracy.

Although most studies relied on handcrafted radiomic features, a growing tendency to use DL models or combine DL with handcrafted radiomics is appreciated [18]. DL features are promising as they capture complex patterns, are less sensitive to segmentation and preprocessing variations. Moreover, results show a tendency to achieve higher model AUC values. However, DL models are more complex, prone to overfitting, and less interpretable [37], emphasizing the need for future research to ensure DL model robustness and clinical applicability.

Beyond feature type, incorporating clinical and dosimetric variables may further improve performance. While most studies included clinical variables and showed improved performance, treatment-related factors such as radiation dose were considered in only three studies [32, 52, 53], highlighting an underexplored opportunity to further enhance predictive accuracy.

Class imbalance scenarios, as well as dimensionality reduction processes and data partitioning, are factors that introduce challenges during model development. Currently, there is no consensus regarding the methods used to address these challenges. The most common techniques are SMOTE for class imbalance, mRMR and LASSO for dimensionality reduction, and k-fold cross-validation and bootstrap for data partitioning.

A variety of algorithms were used across studies, with RF and SVM being the most frequently used, probably for their simple but optimal performance in small and high-dimensional datasets. More recent studies introduce more complex architectures (DL), often achieving higher AUC values, but mostly tested in small cohorts and less-quality studies, increasing the risk of overfitting. So, results should be interpreted cautiously. Notably, high-quality studies with larger sample sizes tended to rely on classical ML combined with rigorous feature reduction rather than complex DL models, suggesting that methodological robustness currently contributes more to model performance than algorithm complexity.

Quality assessments revealed low to moderate methodological standards, with a steady improvement in recent years. The mean RQS score was modest, with persistent gaps reflecting both methodological limitations and practices that remain uncommon in radiomics research. Particularly, we highlight the inability to compare radiomic results against a gold-standard procedure, as biopsy of metastases is rarely performed in clinical practice. METRICS scores were generally higher and appeared better suited to evaluate the quality of radiomic studies addressing early ART response in metastases. Recent studies achieved robust METRICS evaluations, indicating stronger reporting transparency, internal validation, and methodological rigor. However, aspects crucial for clinical translation, such as multicentric design, model accessibility, and generalizability, remain underdeveloped.

Applying multiple quality frameworks showed that high RQS scores do not necessarily align with high METRICS scores, consistent with Kocak et al [58], reflecting the distinct aspects each tool evaluates: RQS emphasizes biological validity, clinical relevance, and methodological rigor, whereas METRICS focuses on transparent reporting, reproducibility, and AI model development. Importantly, METRICS offers an updated framework tailored to current radiomics and DL studies, addressing several limitations of RQS. However, certain RQS elements not fully captured by METRICS, such as “phantom study,” “multiple time points,” “biological correlates,” and “prospective study,” may remain relevant when evaluating studies linking imaging patterns with biological or longitudinal processes.

Overall, radiomics shows promise for early prediction of metastatic ART response and personalized care. Future studies should prioritize larger, multicentric and adequately powered cohorts, with a special focus on extracranial lesions. Imaging standardization and careful assessment of the impact of different methodologies across the radiomic pipeline (image acquisition and preprocessing, feature extraction, ROI definition and model construction) remains crucial. Efforts should also focus on strategies to limit or assess overfitting, manage data scarcity, and improve model interpretability. Integrating multimodal variables, such as clinical and dosimetric data, as well as emerging approaches like radiogenomics and metabolic imaging [59–61], may further enhance models’ predictive performance. Following METRICS and CLEAR frameworks, while considering relevant RQS elements, will strengthen methodological quality, reproducibility and clinical translation, ultimately supporting personalized oncological treatments and improving patient outcomes.

## Conclusion

Radiomics-based predictive models hold strong potential to anticipate treatment response in metastases treated with ART. Yet, evidence remains preliminary, particularly for bone, lung, and liver lesions. Methodological quality has improved in recent studies; however, clinical translation requires prospective, multicenter validation, standardized imaging protocols, and the inclusion of clinical and dosimetric variables. Achieving these goals will enable radiomic biomarkers to evolve from exploratory tools into reliable instruments that support personalized radiotherapy and improve patient outcomes.

## Supplementary information

ELECTRONIC SUPPLEMENTARY MATERIAL

Supplementary Table 2-6

## Data Availability

The data that support the findings of this study are included in the article and the supplementary materials.

## Abbreviations

AI: Artificial intelligence
ART: Ablative radiotherapy
AUC: Area under the curve
CLEAR: Checklist for evaluation of radiomics research
CNN: Convolutional neural network
DL: Deep learning
KPS: Karnofsky performance status
LINAC: Linear accelerator
METRICS: Methodological radiomics score
ML: Machine learning
OMD: Oligometastatic disease
PICO: Patient, operation, comparator, and outcome
RANO-BM: Response assessment in neuro-oncology brain metastases
RECIST: Response evaluation criteria in solid tumors
RF: Random forest
ROC: Receiver operating characteristics
RQS: Radiomics quality score
SABR: Stereotactic ablative radiotherapy
SRS: Stereotactic radiosurgery
SRT: Stereotactic radiotherapy
SVM: Support vector machine
