# Supplementary material for: Predicting early response to ablative radiotherapy in oligometastatic disease: a scoping review of radiomics-based machine learning and deep learning models
Source: Eur Radiol. 2026 Apr 30;36(9):6997–7009. doi: 10.1007/s00330-026-12590-9 (PMC13451257; doi:10.1007/s00330-026-12590-9)
Supplement: Supplementary file 1 [file 330_2026_12590_MOESM1_ESM.pdf]

Predicting early response to ablative radiotherapy in oligometastatic disease: A scoping review of radiomics-based machine learning and deep learning models

ELECTRONIC SUPPLEMENTARY MATERIAL

Supplementary Table 1.
Complete search strategies in the different electronic databases.

Database	Search strategy
**PubMed**	#1 ((brain neoplasm[MeSH Terms]) OR (brain neoplasm[Title/Abstract])) OR (((((((((((((((brain[Title/Abstract]) OR (brain[MeSH Terms])) OR (encephalon[Title/Abstract])) OR (central nervous system[Title/Abstract])) OR (cns[Title/Abstract])) OR (cerebral[Title/Abstract])) OR (cerebell*[Title/Abstract])) OR (brain stem[Title/Abstract])) OR (intracranial[Title/Abstract])) AND (neoplasm metastasis[MeSH Terms])) OR (neoplasm metastas*)) OR (neoplasm metastas*[Title/Abstract])) OR (oligometastas*[Title/Abstract])) OR (metastas*[Title/Abstract])) OR (metastas*))
	#2 ((((((((((lung) OR (lung[MeSH Terms])) OR (lung[Title/Abstract])) OR (pulmonary)) OR (pulmonary[Title/Abstract])) AND (neoplasm metastasis[MeSH Terms])) OR (Neoplasm Metastases)) OR (neoplasm metastasis[Title/Abstract])) OR (oligometastas*[Title/Abstract])) OR (metastas*)) OR (metastas*[Title/Abstract])
	#3 (((((((((liver) OR (liver[MeSH Terms])) OR (liver[Title/Abstract])) OR (hepatic)) OR (hepatic[Title/Abstract])) AND (Neoplasm Metastases)) OR (neoplasm metastas*[Title/Abstract])) OR (oligometastas*[Title/Abstract])) OR (metastas*)) OR (metastas*[Title/Abstract])
	#4 (((((((((((((((((((((bone) OR (bone[MeSH Terms])) OR (bone[Title/Abstract])) OR (bones[Title/Abstract])) OR (bone tissue[Title/Abstract])) OR (skeleton)) OR (skeleton[Title/Abstract])) OR (skeletal)) OR (skeletal[Title/Abstract])) OR (osseous)) OR (osseous[Title/Abstract])) OR (bony[Title/Abstract])) OR (spine[Title/Abstract])) OR (spinal[Title/Abstract])) OR (osteolytic metastas*[Title/Abstract])) OR (osteoblastic metastas*[Title/Abstract])) AND (neoplasm metastases)) OR (neoplasm metastases[MeSH Terms])) OR (neoplasm metastas*[Title/Abstract])) OR (oligometastas*[Title/Abstract])) OR (metastas*[Title/Abstract])) OR (metastas*)
	#5 ((((((((((((((((((radiotherapy[MeSH Terms]) OR (radiotherapy)) OR (radiotherapy[Title/Abstract])) OR (radiation therapy[Title/Abstract])) OR (targeted radiotherapy[Title/Abstract])) OR (radiosurgery[MeSH Terms])) OR (radiosurgery[Title/Abstract])) OR (cyberknife radiosurgery[Title/Abstract])) OR (gamma knife radiosurgery[Title/Abstract])) OR (LINAC radiosurgery[Title/Abstract])) OR (linear accelerator radiosurgery[Title/Abstract])) OR (stereotactic body radiotherapy[Title/Abstract])) OR (stereotactic radiation[Title/Abstract])) OR (stereotactic radiation therapy[Title/Abstract])) OR (stereotactic radiosurgery[Title/Abstract])) OR (sabr[Title/Abstract])) OR (sbrt[Title/Abstract])) OR (stereotactic ablative radiotherapy[Title/Abstract])) OR (stereotactic body radiation therapy[Title/Abstract])
	#6 (((((((((((((((((((radiomics[MeSH Terms]) OR (radiomic*[Title/Abstract])) OR (Artificial Intelligence[MeSH Terms])) OR (Artificial Intelligence[Title/Abstract])) OR (ai[Title/Abstract])) OR (Neural networks computer[Title/Abstract])) OR (Machine Learning[MeSH Terms])) OR (Machine Learning[Title/Abstract])) OR (Deep Learning[MeSH Terms])) OR (Deep Learning[Title/Abstract])) OR (Supervised Machine Learning[MeSH Terms])) OR (Supervised Machine Learning[Title/Abstract])) OR (Unsupervised Machine Learning[MeSH Terms])) OR (Unsupervised Machine Learning[Title/Abstract])) OR (Support Vector Machine[MeSH Terms])) OR (Support Vector Machine[Title/Abstract])) OR (svm[Title/Abstract])) OR (conventional neural network[Title/Abstract])) OR (convolutional neural network[Title/Abstract])) OR (artificial neural network*[Title/Abstract])
	#1 OR #2 OR #3 #4 AND #5 AND #6
**Web of Science**	(((((("brain neoplasm") OR ("brain neoplasm")) OR ((((((((((((((brain) OR (brain)) OR (encephalon)) OR ("central nervous system")) OR (cns)) OR (cerebral)) OR (cerebell*)) OR ("brain stem")) OR (intracranial)) AND ("neoplasm metastasis")) OR ("neoplasm metastas*")) OR ("neoplasm metastas*")) OR (oligometastas*)) OR (metastas*)) OR (metastas*))) OR ((((((((((lung) OR (lung)) OR (lung)) OR (pulmonary)) OR (pulmonary)) AND ("neoplasm metastasis")) OR ("Neoplasm Metastases")) OR ("neoplasm metastasis")) OR (oligometastas*)) OR (metastas*)) OR (metastas*))) OR ((((((((((((((((((((((bone) OR (bone)) OR (bone)) OR (bones)) OR ("bone tissue")) OR (skeleton)) OR (skeleton)) OR (skeletal)) OR (skeletal)) OR (osseous)) OR (osseous)) OR (bony)) OR (spine)) OR (spinal)) OR ("osteolytic metastas*")) OR ("osteoblastic metastas*")) AND ("neoplasm metastases")) OR ("neoplasm metastases")) OR ("neoplasm metastas*")) OR (oligometastas*)) OR (metastas*)) OR (metastas*))) AND (((((((((((((((((((radiotherapy) OR (radiotherapy)) OR (radiotherapy)) OR ("radiation therapy")) OR ("targeted radiotherapy")) OR (radiosurgery)) OR (radiosurgery)) OR ("cyberknife radiosurgery")) OR ("gamma knife radiosurgery")) OR ("LINAC radiosurgery")) OR ("linear accelerator radiosurgery")) OR ("stereotactic body radiotherapy")) OR ("stereotactic radiation")) OR ("stereotactic radiation therapy")) OR ("stereotactic radiosurgery")) OR (sabr)) OR (sbrt)) OR ("stereotactic ablative radiotherapy")) OR ("stereotactic body radiation therapy"))) AND ((((((((((((((((((((radiomics) OR (radiomic*)) OR ("Artificial Intelligence")) OR ("Artificial Intelligence")) OR (ai)) OR ("Neural networks computer")) OR ("Machine Learning")) OR ("Machine Learning")) OR ("Deep Learning")) OR ("Deep Learning")) OR ("Supervised Machine Learning")) OR ("Supervised Machine Learning")) OR ("Unsupervised Machine Learning")) OR ("Unsupervised Machine Learning")) OR ("Support Vector Machine")) OR ("Support Vector Machine")) OR (svm)) OR ("conventional neural network")) OR ("convolutional neural network")) OR ("artificial neural network*"))
**Scopus**	(((((("brain neoplasm") OR ("brain neoplasm")) OR ((((((((((((((brain) OR (brain)) OR (encephalon)) OR ("central nervous system")) OR (cns)) OR (cerebral)) OR (cerebell*)) OR ("brain stem")) OR (intracranial)) AND ("neoplasm metastasis")) OR ("neoplasm metastas*")) OR ("neoplasm metastas*")) OR (oligometastas*)) OR (metastas*)) OR (metastas*))) OR ((((((((((lung) OR (lung)) OR (lung)) OR (pulmonary)) OR (pulmonary)) AND

	("neoplasm metastasis")) OR ("Neoplasm Metastases")) OR ("neoplasm metastasis")) OR (oligometastas*)) OR (metastas*)) OR (metastas*))) OR ((((((((((((((((((((((bone) OR (bone)) OR (bone)) OR (bones)) OR ("bone tissue")) OR (skeleton)) OR (skeleton)) OR (skeletal)) OR (skeletal)) OR (osseous)) OR (osseous)) OR (bony)) OR (spine)) OR (spinal)) OR ("osteolytic metastas*")) OR ("osteoblastic metastas*")) AND ("neoplasm metastases")) OR ("neoplasm metastases")) OR ("neoplasm metastas*")) OR (oligometastas*)) OR (metastas*)) OR (metastas*))) AND (((((((((((((((((((radiotherapy) OR (radiotherapy)) OR (radiotherapy)) OR ("radiation therapy")) OR ("targeted radiotherapy")) OR (radiosurgery)) OR (radiosurgery)) OR ("cyberknife radiosurgery")) OR ("gamma knife radiosurgery")) OR ("LINAC radiosurgery")) OR ("linear accelerator radiosurgery")) OR ("stereotactic body radiotherapy")) OR ("stereotactic radiation")) OR ("stereotactic radiation therapy")) OR ("stereotactic radiosurgery")) OR (sabr)) OR (sbrt)) OR ("stereotactic ablative radiotherapy")) OR ("stereotactic body radiation therapy"))) AND ((((((((((((((((((((radiomics) OR (radiomic*)) OR ("Artificial Intelligence")) OR ("Artificial Intelligence")) OR (ai)) OR ("Neural networks computer")) OR ("Machine Learning")) OR ("Machine Learning")) OR ("Deep Learning")) OR ("Deep Learning")) OR ("Supervised Machine Learning")) OR ("Supervised Machine Learning")) OR ("Unsupervised Machine Learning")) OR ("Unsupervised Machine Learning")) OR ("Support Vector Machine")) OR ("Support Vector Machine")) OR (svm)) OR ("conventional neural network")) OR ("convolutional neural network")) OR ("artificial neural network*"))
**Embase Ovid**	(((((('brain tumor'/exp) OR ('brain neoplasm':ti,ab,kw)) OR (((((((((((((('brain':ti,ab,kw) OR ('brain'/exp)) OR ('encephalon':ti,ab,kw)) OR ('central nervous system':ti,ab,kw)) OR ('cns':ti,ab,kw)) OR ('cerebral':ti,ab,kw)) OR ('cerebell*':ti,ab,kw)) OR ('brain stem':ti,ab,kw)) OR ('intracranial':ti,ab,kw)) AND ('metastasis'/exp)) OR ('neoplasm metastas*')) OR ('neoplasm metastas*':ti,ab,kw)) OR ('oligometastas*':ti,ab,kw)) OR ('metastas*':ti,ab,kw)) OR ('metastas*'))) OR ((((((((((('lung') OR ('lung'/exp)) OR ('lung':ti,ab,kw)) OR ('pulmonary')) OR ('pulmonary':ti,ab,kw)) AND ('metastasis'/exp)) OR ('neoplasm metastases')) OR ('neoplasm metastasis':ti,ab,kw)) OR ('oligometastas*':ti,ab,kw)) OR ('metastas*')) OR ('metastas*':ti,ab,kw))) OR ((((((((((((((((((((('bone') OR ('bone'/exp)) OR ('bone':ti,ab,kw)) OR ('bones':ti,ab,kw)) OR ('bone tissue':ti,ab,kw)) OR ('skeleton')) OR ('skeleton':ti,ab,kw)) OR ('skeletal')) OR ('skeletal':ti,ab,kw)) OR ('osseous')) OR ('osseous':ti,ab,kw)) OR ('bony':ti,ab,kw)) OR ('spine':ti,ab,kw)) OR ('spinal':ti,ab,kw)) OR ('osteolytic metastas*':ti,ab,kw)) OR ('osteoblastic metastas*':ti,ab,kw)) AND ('neoplasm metastases')) OR ('neoplasm metastases'/exp)) OR ('neoplasm metastas*':ti,ab,kw)) OR ('oligometastas*':ti,ab,kw)) OR ('metastas*':ti,ab,kw)) OR ('metastas*'))) AND (((((((((((((((((('radiotherapy'/exp) OR ('radiotherapy')) OR ('radiotherapy':ti,ab,kw)) OR ('radiation therapy':ti,ab,kw)) OR ('targeted radiotherapy':ti,ab,kw)) OR ('radiosurgery'/exp)) OR ('radiosurgery':ti,ab,kw)) OR ('cyberknife radiosurgery':ti,ab,kw)) OR ('gamma knife radiosurgery':ti,ab,kw)) OR ('linac radiosurgery':ti,ab,kw)) OR ('linear accelerator radiosurgery':ti,ab,kw)) OR ('stereotactic body radiotherapy':ti,ab,kw)) OR ('stereotactic radiation':ti,ab,kw)) OR ('stereotactic radiation therapy':ti,ab,kw)) OR ('stereotactic radiosurgery':ti,ab,kw)) OR ('sabr':ti,ab,kw)) OR ('sbrt':ti,ab,kw)) OR ('stereotactic ablative radiotherapy':ti,ab,kw)) OR ('stereotactic body radiation therapy':ti,ab,kw))) AND (((((((((((((((((('radiomics'/exp) OR ('radiomic*':ti,ab,kw)) OR ('artificial intelligence'/exp)) OR ('artificial intelligence':ti,ab,kw)) OR ('ai':ti,ab,kw)) OR ('neural networks computer':ti,ab,kw)) OR ('machine learning'/exp)) OR ('machine learning':ti,ab,kw)) OR ('deep learning'/exp)) OR ('deep learning':ti,ab,kw)) OR ('supervised machine learning'/exp)) OR ('supervised machine learning':ti,ab,kw)) OR ('unsupervised machine learning'/exp)) OR ('unsupervised machine learning':ti,ab,kw)) OR ('support vector machine'/exp)) OR ('support vector machine':ti,ab,kw)) OR ('svm':ti,ab,kw)) OR ('conventional neural network':ti,ab,kw)) OR ('convolutional neural network':ti,ab,kw)) OR ('artificial neural network*':ti,ab,kw))
**Cochrane Library**	#1 MeSH descriptor: [Brain Neoplasms] explode all trees 3160<br>#2 (brain metastas*):ti,ab,kw 3428<br>#3 MeSH descriptor: [Radiotherapy] explode all trees 9836<br>#4 (radiotherapy):ti,ab,kw 42941<br>#5 MeSH descriptor: [Radiomics] explode all trees 66<br>#6 (radiomic*):ti,ab,kw 790<br>#7 #1 OR #2 5817<br>#8 #3 OR #4 43752<br>#9 #5 OR #6 790<br>#10 #7 AND #8 AND #9 9<br><br>#1 MeSH descriptor: [Lung] explode all trees 6288<br>#2 MeSH descriptor: [Neoplasm Metastasis] explode all trees 7602<br>#3 (lung):ti,ab,kw 92177<br>#4 (metastas*):ti,ab,kw 33411<br>#5 MeSH descriptor: [Radiotherapy] explode all trees 9836<br>#6 (radiotherapy):ti,ab,kw 42941<br>#7 MeSH descriptor: [Radiomics] explode all trees 66<br>#8 (radiomic*):ti,ab,kw 790<br>#9 #1 OR #3 AND #2 OR #4 39675<br>#10 #5 OR #6 43752<br>#11 #7 OR #8 790<br>#12 #9 AND #10 AND #11 22<br><br>#1 MeSH descriptor: [Lung] explode all trees 6288

	#2 MeSH descriptor: [Neoplasm Metastasis] explode all trees 7602<br>#3 (lung):ti,ab,kw 92177<br>#4 (metastas*):ti,ab,kw 33411<br>#5 MeSH descriptor: [Radiotherapy] explode all trees 9836<br>#6 (radiotherapy):ti,ab,kw 42941<br>#7 MeSH descriptor: [Radiomics] explode all trees 66<br>#8 (radiomic*):ti,ab,kw 790<br>#9 #1 OR #3 AND #2 OR #4 39675<br>#10 #5 OR #6 43752<br>#11 #7 OR #8 790<br>#12 #9 AND #10 AND #11 22<br>#13 MeSH descriptor: [Liver] explode all trees 4649<br>#14 (liver):ti,ab,kw 68592<br>#15 #13 OR #14 AND #2 OR #4 37962<br>#16 #15 AND #10 AND #11 21<br><br>#1 MeSH descriptor: [Lung] explode all trees 6288<br>#2 MeSH descriptor: [Neoplasm Metastasis] explode all trees 7602<br>#3 (lung):ti,ab,kw 92177<br>#4 (metastas*):ti,ab,kw 33411<br>#5 MeSH descriptor: [Radiotherapy] explode all trees 9836<br>#6 (radiotherapy):ti,ab,kw 42941<br>#7 MeSH descriptor: [Radiomics] explode all trees 66<br>#8 (radiomic*):ti,ab,kw 790<br>#9 #1 OR #3 AND #2 OR #4 39675<br>#10 #5 OR #6 43752<br>#11 #7 OR #8 790<br>#12 #9 AND #10 AND #11 22<br>#13 MeSH descriptor: [Liver] explode all trees 4649<br>#14 (liver):ti,ab,kw 68592<br>#15 #13 OR #14 AND #2 OR #4 37962<br>#16 #15 AND #10 AND #11 21<br>#17 MeSH descriptor: [Bone and Bones] explode all trees 18843<br>#18 (bone):ti,ab,kw 71072<br>#19 #17 OR #18 AND #2 OR #4 52153<br>#20 #19 AND #10 AND #11 21
**Google Scholar**	1. “Brain metastases” AND "SABR" OR "SBRT" OR "SRS" AND "radiomics" 1090<br>2. “Lung metastases” AND "SABR" OR "SBRT" OR "SRS" AND "radiomics" 314<br>3. “Liver metastases” AND "SABR" OR "SBRT" OR "SRS" AND "radiomics" 521<br>4. “Bone metastases” AND "SABR" OR "SBRT" OR "SRS" AND "radiomics" 364
